# Biological Insights Into Muscular Strength: Genetic Findings in the UK Biobank

**DOI:** 10.1038/s41598-018-24735-y

**Published:** 2018-04-24

**Authors:** Emmi Tikkanen, Stefan Gustafsson, David Amar, Anna Shcherbina, Daryl Waggott, Euan A. Ashley, Erik Ingelsson

**Affiliations:** 1 Division of Cardiovascular Medicine, Department of Medicine, Stanford University School of Medicine, Stanford, CA USA; 2 Department of Medical Sciences, Molecular Epidemiology and Science for Life Laboratory, Uppsala University, Uppsala, Sweden; 3 Stanford Cardiovascular Institute, Stanford University, Stanford, CA 94305 USA

## Abstract

We performed a large genome-wide association study to discover genetic variation associated with muscular strength, and to evaluate shared genetic aetiology with and causal effects of muscular strength on several health indicators. In our discovery analysis of 223,315 individuals, we identified 101 loci associated with grip strength (*P* <5 × 10^−8^). Of these, 64 were associated (*P* < 0.01 and consistent direction) also in the replication dataset (N = 111,610). eQTL analyses highlighted several genes known to play a role in neuro-developmental disorders or brain function, and the results from meta-analysis showed a significant enrichment of gene expression of brain-related transcripts. Further, we observed inverse genetic correlations of grip strength with cardiometabolic traits, and positive correlation with parents’ age of death and education. We also showed that grip strength had shared biological pathways with indicators of frailty, including cognitive performance scores. By use of Mendelian randomization, we provide evidence that higher grip strength is protective of both coronary heart disease (OR = 0.69, 95% CI 0.60–0.79, P < 0.0001) and atrial fibrillation (OR = 0.75, 95% CI 0.62–0.90, P = 0.003). In conclusion, our results show shared genetic aetiology between grip strength, and cardiometabolic and cognitive health; and suggest that maintaining muscular strength could prevent future cardiovascular events.

## Introduction

Hand grip strength is a simple and non-invasive measurement of general muscular strength and it has been shown to predict disability in older adults, fracture risk, nutritional status, cardiovascular disease events and all-cause mortality^1–3^. Several behavioral and environmental factors, such as physical activity and nutrition, affect the variability of grip strength, but family studies have suggested that genetic factors also have a significant role^4,5^, with estimated 56% heritability^6^. The identification of genetic variants affecting grip strength variability could help in the understanding of biological mechanisms of muscular fitness, as well as lend biological insights to physical functioning late in life and healthy aging.

So far, two genome-wide association studies (GWAS) of maximal grip strength have been conducted^7,8^. The largest study, also conducted in the UK Biobank, included 195,180 individuals and identified 16 loci associated with grip strength. In this study, we conducted traditional and gene-based GWAS for 334,925 individuals from the UK Biobank to discover novel loci for relative grip strength^9,10^, and evaluated shared genetic aetiology and causal effects of grip strength on several health indicators.

## Results

### Genetic associations for grip strength in biologically relevant loci

**Figure 1:**
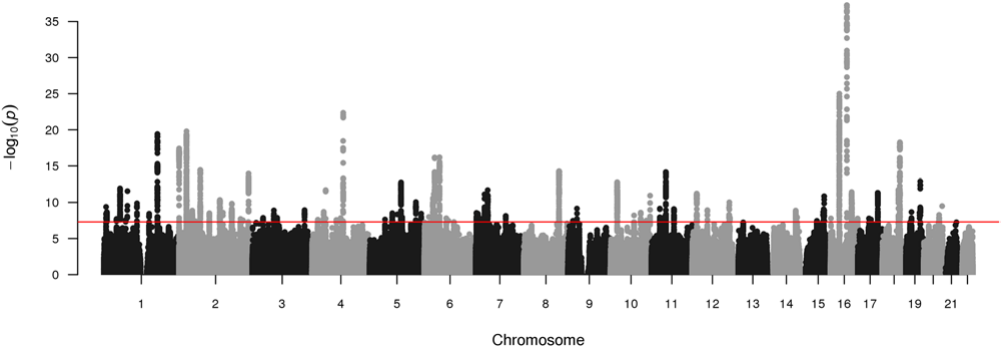
Manhattan plot of genetic associations with grip strength in discovery sample.

In our discovery GWAS (random 2/3 sample from eligible individuals; N = 223,315) adjusted for age, sex, genotype array, and 10 principal components, we identified 101 genome-wide significant loci for grip strength (Fig. 1). Four variants were independent single-nucleotide polymorphisms (SNPs) from loci with another lead variant, identified through conditional analyses (rs62106258 near *LINC01874*, rs78648104 in *TFAP*2*D*, rs800895 in *TRPS1* and rs10871777 near *ENSG00000267620*). Out of 101 variants, 64 were associated (P < 0.01 and consistent direction) in the replication dataset (remaining 1/3 of eligible individuals; N = 111,610; Supplementary Table S1). Most of the 64 replicating SNPs were located in introns (48%) or in intergenic regions (22%), while only 8% were located in exons. The corresponding proportions across all SNPs were 57%, 28% and 1% (for intronic, intergenic and exonic SNPs, respectively). Thus, exonic SNPs were replicated at higher rate compared to intronic and intergenic SNPs, which has been suggested in some prior literature^11^.

**Figure 2:**
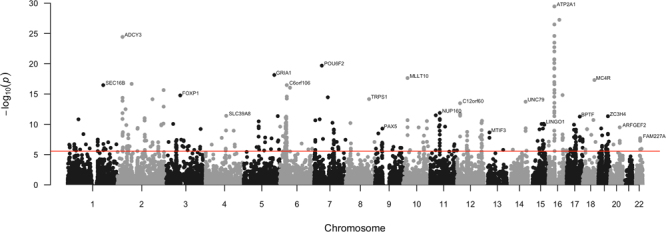
Gene-based manhattan plot of genetic associations with grip strength in discovery sample. The most significant gene for each chromosome is labeled.

The two loci with most significant associations were located in chromosome 16, in the intron of *FTO* (rs1421085, β = −0.004, P = 5.4 × 10^−38^ and β = −0.004, P = 4.9 × 10^−22^ in discovery and replication samples, respectively) and *ATXN2L* (rs12928404, β = −0.003, P = 1.0 × 10^−25^ and β = −0.002, P = 1.1 × 10^−7^). The *FTO* locus has been previously shown to be associated with obesity^12,13^ and lipids^14^, among other metabolic traits; and according to the OMIM database, mutations in this gene can also cause growth retardation, developmental delay, and facial dysmorphism. The other locus on chromosome 16 has been reported to be associated with intelligence in a recent large study^15^, and our lead variant has a high probability of being regulatory (Regulome score^16^ = 1b). This is also in close vicinity of *ATP2A1*, a gene involved in muscular contraction and relaxation, and a causal gene for a muscle disorder called Brody disease, which is characterized by muscle cramping after exercise. Our gene-based analysis of the discovery sample identified *ATP2A1* as the most significant gene for grip strength (P = 3.9 × 10^−31^, Fig. 2). Among the top three most significant loci was a nonsynonymous SNP in the exon of *SLC39A8* (rs13107325, β = −0.006, P = 4.4 × 10^−23^ and β = −0.005, P = 2.0 × 10^−10^), which has been identified as a susceptibility variant for schizophrenia^17^ and metabolic traits^12,18^. This variant had a CADD score^19^ of 34.00, predicting deleterious effect, and mutations in *SLC39A8* are also known to cause a severe congenital disorder of glycosylation, characterized by delayed psychomotor development apparent from infancy, hypotonia, short stature, seizures, visual impairment, and cerebellar atrophy.

To identify candidate genes regulated by the 64 replicated grip strength variants, we used the Genotype-Tissue Expression (GTEx) database^20^ for eQTL analyses across all tissues. In 25 loci, we found evidence of at least one significant eQTL (FDR ≤ 0.05, Supplementary Table S2). The largest number of significant eQTLs was associated with gene expression in nerve (tibial), artery (tibial), and skin (sun-exposed lower leg). In seven loci, the most significant eQTL was found for the gene in which they were located (*ADCY3*, *TGFA*, *BDNF*, *KIF1B*, *LRRC43*, *ARPP21*, *KIAA1598*). Our top SNP in *BDNF* (rs6265, β = 0.002, P = 7.1 × 10^−10^), is located in the exon and has a high CADD score (CADD = 24.1), highlighting that this is likely to be a pathogenic variant. This is an interesting gene as it encodes brain-derived neurotrophic factor, an important growth factor promoting neurogenesis. BDNF concentrations are increased in response to exercise and decreased in neurodegenerative diseases^21^. Mutations in *BDNF* might also cause congenital central hypoventilation syndrome, characterized by hypoventilation due to the absence of primary neuromuscular, lung, or cardiac disease, or an identifiable brainstem lesion. *KIF1B* is another interesting gene involved in Charcot-Marie-Tooth disease, which is characterized by distal limb muscle weakness and atrophy due to peripheral neuropathy. *TGFA* encodes a growth factor that activates a signaling pathway for cell proliferation, differentiation and development, and OMIM links include cancers, cleft lip and Alstrom syndrome.

Interestingly, some variants regulated the expression of other genes having a role in developmental abnormalities. The most significant eQTL was one of the lead variants, rs12928404 in *ATXN2L*, which had a significant association with the expression of adjacent *TUFM* gene in several tissues (lowest P = 5.2 × 10^−69^, FDR = 0.0003 for whole blood). Mutations in *TUFM* have previously been shown to cause combined oxidative phosphorylation deficiency 4, a syndrome consisting of intrauterine growth retardation, developmental regression, hypotonia and respiratory failure. Another interesting eQTL was rs6759321, which regulated *DARS* expression in thyroid (P = 5.8 × 10^−7^, FDR = 0.0002). *DARS* is a causal gene for another disorder including delayed motor development, mental retardation, among other features (“hypomyelination with brainstem and spinal cord involvement and leg spasticity”^22^). Further, rs12599952 regulated the expression of *DHODH* in several tissues (lowest P = 1.6 × 10^−11^, FDR = 0.0003 for tibial artery), a gene responsible for Miller syndrome including severe micrognathia, cleft lip and/or palate, hypoplasia, eyelid coloboma, and accessory nipples and several other developmental abnormalities.

Finally, several genes located nearest to the lead variants (which generally does not demonstrate causality, but sometimes can be indicative of the mechanisms) are also known to play roles in different developmental disorders (Supplementary Table S2). These include patent ductus arteriosus, a form of congenital heart defect (*TFAP2B*), myotonic dystrophy type 1, which is the most prevalent adult onset muscular dystrophy (*CELF1*), Pitt-Hopkins syndrome, which is characterized by intellectual disability, distinctive facial features, poor muscular development and abnormal breathing (*TCF4*), mental retardation with language impairment and with or without autistic features (*FOXP1*) and hyaline fibromatosis syndrome, characterized by abnormal growth of hyalinized fibrous tissue (*ANTXR2*).

### Genetic risk score analysis

 Next, we calculated a genetic risk score (GRS) as a weighted sum of the 101 grip strength variants identified in the discovery dataset and estimated its associations with the measures of fitness, general health and indicators of frailty^23^ in the replication dataset (Table 1). The GRS was significantly associated with cardiorespiratory fitness (V̇O_2_, N = 14,681), objective measurement of physical activity (average acceleration measured with a wrist-worn accelerometer, N = 24,282), self-reported good or excellent overall health (N = 112,498), and fluid intelligence score (N = 36,366). The significant inverse associations were observed with slow walking speed (N = 112,498), frequent feelings of tiredness / lethargy in last 2 weeks (N = 112,498), falls during the last year (N = 112,498), weight loss during the last year (N = 112,498), and reaction time (N = 111,812). All association remained significant after multiple testing correction (alpha threshold = 0.05/9).

**Table 1:** Association between genetic risk score for grip strength and frailty indices.

Trait	Beta^*^	Se	P	N
Physical activity (accelerometer)	0.018	0.006	0.003	24282
Cardiorespiratory fitness (V̇O_2_)	0.201	0.022	3.1 × 10^−19^	14681
General Health	0.068	0.007	1.3 × 10^−22^	112498
Slow walking speed	−0.106	0.011	1.1 × 10^−20^	112498
Falls	−0.041	0.008	8.5 × 10^−8^	112498
Weight loss	−0.067	0.008	9.9 × 10^−16^	112498
Tiredness	−0.026	0.006	1.4 × 10^−5^	112498
Fluid intelligence score	0.014	0.005	0.0046	36366
Reaction time	−0.012	0.003	1.6 × 10^−5^	111812

^*^Per SD in genetic risk score and outcome variable. Genetic risk score was calculated as a sum of grip strength increasing alleles, weighted with the effect sizes from discovery analysis. Associations with frailty indices were tested in the replication sample. V̇O_2_: net oxygen consumption.

### Meta-analysis

To maximize power for pathway and Mendelian randomization (MR) analyses and to suggest additional associations, we also performed a meta-analysis of the discovery and replication samples. This analysis revealed 139 independent loci reaching genome-wide significance for grip strength (r2 = 0.05, clumping window = 500 kb, Supplementary Table S3). These variants explained 1.7% of the grip strength variance. In a sensitivity analysis, we tested for associations between the effects of these 139 SNPs on our definition of grip strength and the effects on alternative measures of grip strength (grip strength divided by BMI, as well as maximum grip strength). The correlations between the effect sizes were high (r = 0.99 for grip strength divided by BMI, r = 0.79 for maximal grip strength). Based on LD-score regression of meta-analyzed results, the genome-wide “chip” heritability of grip strength was 0.13 (SE = 0.004). This is lower than the heritability reported in a recent GWAS study of maximum grip strength (0.24, SE = 0.027)^8^. This discrepancy is likely due to a different phenotype definition; we used relative instead of absolute grip strength to reduce confounding effect of body size (which also may have driven up the heritability estimate of the prior study somewhat). We observed significant inflation of P-values (λ_GC_ = 1.55), but the LD-score regression intercept of 0.9 (SE = 0.01) suggested that this was due to polygenicity, rather than population stratification.

**Figure 3:**
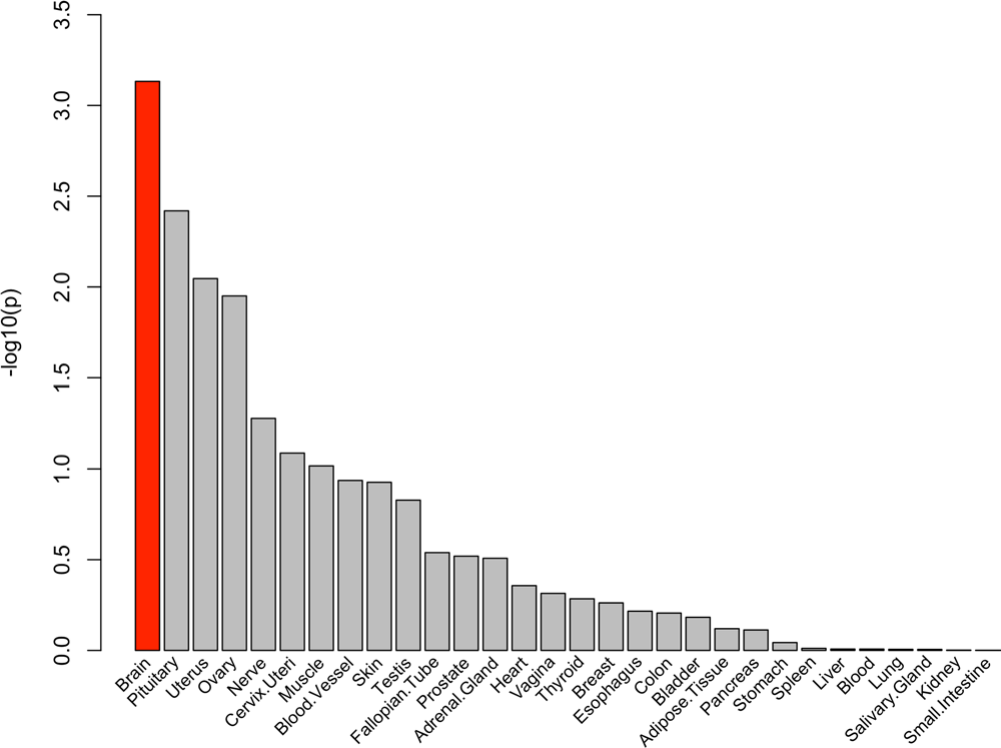
Enrichment of tissue-expression for grip strength loci in 30 tissue types.

Using the full distribution of SNP P-values from the meta-analysis, we observed a significant positive relationship between genes highly expressed in brain and genetic associations for grip strength (Fig. 3). When using only the nearest genes in meta-analysis as input genes, we observed the most significant enrichment of differentially expressed genes in muscle (down-regulated genes P = 0.003, adjusted P = 0.09, N genes in category = 4,396, N overlapping genes = 134). Further, the proportion of overlapping genes in GO biological processes gene sets was highest for “regulation of skeletal muscle contraction” (overlapping genes *GSTM2*, *CASQ1*, *ATP2A1*, P = 6.3 × 10^−5^, adjusted P = 0.009), which is described as any process that modulates the frequency, rate or extent of skeletal muscle contraction.

### Genetic correlations

**Figure 4:**
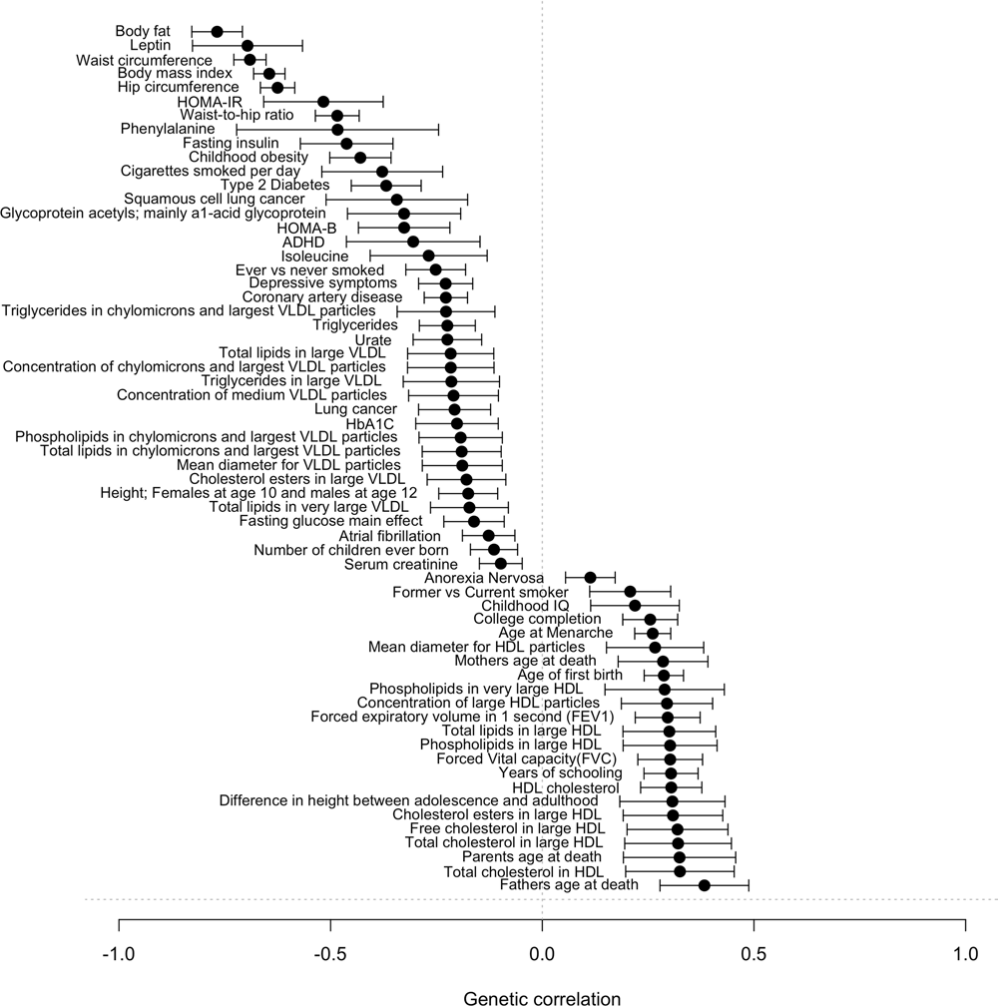
Significant genetic correlations with grip strength. Abbreviations: HOMA-IR, homeostasis model assessment-estimated insulin resistance; HOMA-B, homeostasis model assessment-estimated beta-cell function; ADHD, attention deficit hyperactivity disorder; VLDL, very low density lipoprotein; HbA1c, glycated haemoglobin; IQ, intelligence quotient; HDL, high density lipoprotein.

To evaluate genome-wide heritability (*h*^*2*^_*g*_), partitioned heritability by functional categories, and genetic correlation between other traits, we applied LD-score regression^24–27^ on the results from our meta-analysis. In line with 17 complex diseases and traits analyzed by Finucane *et al*.^27^, we identified strong enrichment of grip strength loci in conserved regions (proportion of heritability / proportion of SNPs = 15.6, P = 5.0 × 10^−20^, FDR = 2.6 × 10^−18^). When testing for genetic correlations between grip strength and all traits in LD Hub^26^, we observed significant genetic correlations with 78 traits (P threshold = 0.05/231, Fig. 4), of which most of them were cardiometabolic traits. The strongest negative correlations were observed with obesity measures and leptin. Interestingly, strong negative correlations were also detected with attention deficit hyperactivity disorder and depressive symptoms. The strongest positive correlations were observed with parent’s age at death, high-density lipoprotein, years of schooling and forced vital capacity.

### Mendelian randomization

We selected 139 SNPs associated with grip strength in the meta-analysis as our genetic instrument and used it to estimate the causal effects of grip strength on coronary heart disease (CHD) and atrial fibrillation (AF). At an alpha level of 0.05, the statistical power to detect a causal effect size of 0.79 (per SD-increase of grip strength; corresponding to the observed association^28^) for grip strength and CHD was 100%. For grip strength and AF, the power was 99% (effect size 0.75^28^). The results from the two-sample MR indicated a causal effect of grip strength on CHD (inverse-variance weighted [IVW]: OR = 0.59, 95% CI 0.49–0.71, P < 0.0001; weighted median: OR = 0.52, 95% CI 0.43–0.63, P < 0.0001, MR Egger: OR = 0.36, 95% CI 0.19–0.69, P = 0.002) and AF (IVW: OR = 0.69, 95% CI 0.57–0.84, P < 0.0001; Weighted median: OR = 0.64, 95% CI 0.49–0.82, P < 0.0001, MR Egger: OR = 0.35, 95% CI 0.17–0.70, P = 0.003). For CHD, our analyses showed no significant horizontal pleiotropy (all P > 0.10). For AF, Egger regression indicated significant pleiotropy (P < 0.05).

As our initial analyses indicated significant heterogeneity of the genetic instruments, we excluded SNPs based on their contribution to a heterogeneity test statistic^29^. This resulted in more consistent causal effect estimates across different methods (CHD: IVW: OR = 0.69, 95% CI 0.60–0.79, P < 0.0001; Weighted median: OR = 0.65, 95% CI 0.54–0.79, P < 0.0001, MR Egger: OR = 0.52, 95% CI 0.31–0.87, P = 0.01; AF: IVW: OR = 0.75, 95% CI 0.62–0.90, P = 0.003; Weighted median: OR = 0.67, 95% CI 0.52–0.86, P = 0.002, MR Egger: OR = 0.40, 95% CI 0.19–0.84, P = 0.02, Supplementary Tables S4-S5). Horizontal pleiotropy was not detected in this analysis restricting our IV to less heterogeneous (and likely less pleiotropic) variants (P > 0.05).

## Discussion

In our GWAS of 223,315 individuals in the discovery and 111,610 in replication sample, we report 64 loci robustly associated with grip strength. The largest number of significant eQTLs was observed for tibial nerve, and our eQTL analyses highlighted several genes known to have a role in neurodevelopmental disorders or brain function. In our meta-analysis of discovery and replication samples, the number of significant loci reached 139 independent variants, and we observed a significant enrichment of gene expression in brain. We observed inverse genetic correlations with cardiometabolic traits, but also attention deficit hyperactivity disorder and depressive symptoms; and positive correlation with parents’ age of death and education. To further assess shared biological pathways with physical and cognitive health, we tested for association between a grip strength GRS and health indicators, including cognitive performance scores, and found that higher GRS was associated with higher levels of self-reported general health, physical activity and fitness, and fluid intelligence score. Inverse associations were observed with reaction time and frailty indicators. Finally, our MR analyses suggest that maintaining muscular strength may prevent future cardiovascular events.

Our results allow us to draw several conclusions. First, it is well known that muscular strength depends not only on the quantity of the involved muscles, but also on the ability of the nervous system to appropriately recruit muscle cells^30^. This is consistent with our results highlighting the role of the brain in regulating muscular strength. Second, the underlying biology of grip strength points to genes with a known role in muscle and brain function, and neurodevelopmental disorders; many of which are characterized by disturbances in motor development and intellectual disability. Third, our GRS analyses suggest shared genetic aetiology between muscular strength and cognitive performance, which is concordant with observational and experimental findings about the beneficial effects of exercise training on brain health^21^. The underlying biological mechanisms could relate to the stimulating effects of exercise in the brain, promoting neuroplasticity. This hypothesis also gets support in an evolutionary context^31^. Finally, the existing literature shows that higher grip strength is associated with lower risk of all-cause and cardiovascular mortality^1^, and incidence of cardiovascular events^28^, which is consistent with our findings of high positive genetic correlation with parents’ age of death, negative correlation with several cardiometabolic traits and causal effects of grip strength on cardiovascular events. These findings suggest that maintaining good strength has a key role in preventing future cardiovascular events, which is in line with previous randomized controlled trials showing favorable effects of resistance training on cardiovascular risk factors^32^.

A main strength of our study is the very large sample size, which enabled us to detect a much larger number of genetic variants compared to previous studies^7,8^. In turn, this resulted in much better power to detect causal effects for cardiovascular events, when compared to a recent GWAS for grip strength^8^. We estimate that with the 16 grip strength variants they analyzed, the power to detect a causal estimate for CHD in their analysis was only 51% (effect size = 0.84 per SD-increase of maximal grip strength in the UK Biobank, R2 = 0.003), while our power was 98.5% for the same effect size. Thus, this highlights that assessing power in MR studies is essential in order to prevent false causal inference. We also used relative grip strength as our phenotype, which is less confounded by body size and more suitable to reflect general muscular fitness than maximal grip strength^9,10,33^.

Our study also has some limitations. First, the response rate in UK Biobank was low (5.5%), and hence, the generalizability of our results is unknown^34^. Second, the measurement accuracy might be limited for some of the traits used in this study. For example, cognitive performance was evaluated with crude measurements of cognitive capacity (fluid intelligence score and reaction time) and cardiorespiratory fitness was measured with a submaximal fitness test, which is less accurate than maximal fitness test. Also, even though grip strength is a commonly used proxy for muscular fitness, it captures mainly upper body strength, especially when measured in sitting position. However, it is highly correlated with knee extension muscle strength (measured as a maximal total strength of left plus right leg, r = 0.77 to 0.81)^35^; and due its feasibility, it is convenient proxy for muscular strength in large samples, which are required in genetic association studies. Third, we note the possibility that the enrichment of genetic correlations with cardiometabolic traits might be biased as they are the most commonly studied traits in GWAS. To partly address this, we applied Bonferroni correction to declare significant genetic correlations, which in this case is overly conservative due to high correlations among the traits. Also, in our analysis, the majority of traits included in genetic correlation analysis were not categorized as “cardiometabolic”, “lipids”, or “glycemic”. Fourth, we used multiple SNPs in our genetic instrument to increase power in our MR analysis, which may increase the risk of including pleiotropic effects. That said, we applied a series of sensitivity analysis and robust MR methods to minimize the effect of pleiotropic SNPs; hence, minimizing the risk of horizontal pleiotropy, which would violate MR assumptions. It is important to note, however, that there may still be vertical pleiotropy (or mediation) that does not violate the assumptions. Finally, our GWAS were conducted in unrelated European samples only to avoid population stratification. Thus, the generalizability to other ethnicities is unknown.

In conclusion, we identified a large number of genetic loci associated with hand grip strength providing insights of biological processes involved in individual variation in muscular strength and providing clues for discovery of new treatments for muscle-related diseases. Our results highlight the key role of the central nervous system in strength performance and the importance of maintaining muscular strength to prevent cardiovascular disease.

## Materials and Methods

### Study sample

The UK Biobank is a large longitudinal cohort study including over 500,000 individuals aged 40–69 years. Participants were enrolled in 22 study centers located in England, Scotland and Wales during 2006–2010. Extensive baseline data on medical history, health behavior, and physical measurements were collected by questionnaires and clinical examination. Participants also provided samples blood, urine and saliva and have agreed to have their future health, including disease events, monitored. The UK Biobank study was approved by the North West Multi-Centre Research Ethics Committee and all participants provided written informed consent to participate in the UK Biobank study. All experiments were performed in accordance with relevant guidelines and regulations. The study protocol is available online^36^.

### Phenotypes

Grip strength was measured in a sitting position using a Jamar J00105 hydraulic hand dynamometer. This measures grip force without movement and adjusts the participant’s hand size. The participants were asked to squeeze the device as hard as they could for three seconds, and the maximum value that was reached during that time was recorded. Both hands were measured in turn (UK Biobank field ID 46 for left and 47 for right hand). Due to its high correlations with body size measurements, relative grip strength (absolute strength corrected for body size) has been suggested to be more accurate measurement of strength. Strength might be higher in obese individuals, but the relative strength (muscle strength per kilogram of body weight) is much lower^9,10,33^. Thus, we calculated relative grip strength as an average of measurements of right and left hand divided by weight (ID 21002)^9,10^. Weight was measured with bioelectrical impedance analysis (BIA) Tanita BC418MA. Objective measurement of physical activity was measured with Axivity AX3 wrist-worn triaxial accelerometer^37^. Cardiorespiratory fitness was measured with the cycle ergometry on a stationary bike (eBike, Firmware v1.7). We calculated net oxygen consumption (VO_2_) according to Swain *et al*.^38^ from individuals’ body weight and maximum workload using the equation V̇O_2_ = 7 + 10.8(workload)/weight. Fluid intelligence score was calculated as a sum of the number of correct answers given to the 13 fluid intelligence questions. Reaction time was determined as mean time to correctly identify matches in snap reaction speed game. The categorical variables of self-reported general health and frailty indicators were recoded to binary variables; overall health: good or excellent, 1, others, 0; slow walking speed: slow pace, 1, others, 0; feelings of tiredness / lethargy in last 2 weeks was defined: more than half of the days or more frequently, 1, others, 0; falls during the last year: one or more falls, 1, others, 0; and weight loss during the last year, yes – lost weight, 1, others, 0.

### Genotypes

Genotyping was performed with the UK BiLEVE and UK Biobank Axiom arrays (Affymetrix Research Services Laboratory, Santa Clara, California, USA) including 807,411 and 825,927 markers, respectively. Initial quality control (QC) was conducted centrally by the UK Biobank, and has been described in detail by Bycroft *et al*.^39^. In short, poor quality genetic markers were detected based on statistical tests for batch effects, plate effects, departures from Hardy-Weinberg Equilibrium (HWE), sex effects, array effects, and discordance. Poor quality samples were identified based on the metrics of missing rate and heterozygosity. After quality control, the data consisted of 488,377 samples (N = 49,950 and 438,427 individuals with the UK BiLEVE and UK Biobank Axiom arrays, respectively) and 805,426 single nucleotide polymorphisms (SNPs), which were the imputed with IMPUTE2 by using both HRC and 1000 Genomes Phase 3 merged with the UK10K haplotype reference panels, so that the HRC was preferred for SNPs present in both panels. In our analysis, we used July 2017 release of the imputed genetic marker data, by excluding genetic markers imputed with the UK10K + 1000 Genomes reference panel due to reported imputation error. We further excluded genetic markers with minor allele count ≤30 and imputation quality <0.8. Thus, the total number of genetic markers included in our analysis was 15,275,733. Further, we included only unrelated individuals in the maximum unrelated subset used for principal component analysis, and with self-reported white British descent at the baseline visit and European/Caucasian ethnicity based on the clustering in principal component analysis. The definitions of these subgroups were done centrally by the UK Biobank^39^, and have been used in multiple prior studies^40–42^.

### Association analysis

The discovery GWAS was carried out in the random sample of 223,315 individuals. Analysis was performed with a linear regression by using PLINK^43^ (version 2.0) assuming an additive model for association between phenotypes and genotype dosages. For top loci, we performed a conditional analysis for a region around the lead SNP to identify additional independent variants; we identified regions containing one or more genome-wide significant SNPs (p ≤  5 × 10^−8^) by screening a window of 500 kb adjacent to the first genome-wide significant SNP on each chromosome. If no additional SNPs were identified, the region was limited to that specific SNP, and screening was continued at the next significant SNP. If additional genome-wide significant SNPs were present in this 500 kb window, the window was expanded with 300 kb from the last SNP, and screened for additional SNPs with p ≤ 5 × 10^−8^ until there were no more genome-wide significant SNPs within the next 300 kb. Within each region, the SNP with lowest p-value was assigned as the index SNP. For each region, conditional association analysis was performed adjusting for all index SNPs found on the chromosome. This was repeated until no SNPs reached p ≤ 5 × 10^−8^ in the conditional analyses. For replication, we used the remaining data of 111,610 individuals. Age, sex, genotype array and 10 principal components were used as covariates. Finally, we conducted an inverse-variance weighted fixed-effect meta-analysis with METAL software^44^ with genomic control correction. In this meta-analysis, independent variants were obtained with linkage disequilibrium pruning (r2 = 0.05, clumping window = 500 kb), rather than conditional analysis.

### Functional annotation

We mapped the genomic positions of all replicated lead SNPs from our discovery analysis and annotated them with the closest gene with ANNOVAR. We used OMIM database to search for known genetic disorders for mapped genes. In addition, we obtained CADD scores^19^, the score of deleteriousness of SNPs predicted by 63 functional annotations, and RegulomeDB scores^16^, representing regulatory functionality of SNPs based on eQTLs and chromatin marks. SNPs were also mapped with tissue-specific gene expression levels by utilizing GTEx database^20^. We searched for significant eQTLs (FDR ≤ 0.05) across all available tissues.

Gene-based association test was computed by MAGMA (v. 1.6)^45^, by mapping SNPs into 18,225 protein-coding genes, and then testing the joint association of all markers in the gene with grip strength with a multiple linear principal components regression. Bonferroni-correction was used to define genome-wide significance (alpha threshold = 0.05/18,225). To evaluate enrichment in tissue-specific gene expression, we tested for association between all genetic associations from meta-analysis and gene expression in a specific tissue types by averaging gene-expression per tissue type. Analysis was conducted for 30 tissue types with MAGMA tissue expression analysis using gene expression data from GTEx. Differentially expressed gene (DEG) sets were obtained for the nearest genes using hypergeometric test, and significant DEG sets were determined using Bonferroni correction for up-regulated, down-regulated and both-sided sets separately. The nearest genes were also used for gene set enrichment analysis, which were obtained from MsigDB (v. 5.2). Multiple test correction was performed for each category.

Analyses were conducted with FUMA platform^46^ using functions SNP2GENE and GENE2FUNC.

### Genetic risk score analyses

We included 101 genome-wide significant SNPs identified in our discovery sample for our GRS analysis (97 from the main and four from conditional analysis). These SNPs explained 1.5% of grip strength variance. The GRS was calculated as a weighted sum of the grip strength increasing alleles, by using the effect sizes from the discovery analysis as weights. We evaluated the association between the GRS and cardiorespiratory fitness, objective measurement of physical activity, fluid intelligence score and reaction time with linear regression models adjusted for age, sex, genotype array and 10 principal components. Both GRS and the outcome variables were rank transformed to normal distribution (mean = 0, SD = 1). The associations between the GRS and self-reported overall health, slow walking speed, frequent feelings of tiredness / lethargy in last 2, falls during the last year, and weight loss during the last year were analyzed with logistic regression models adjusted for age, sex, genotype array and principal components.

### LD-score regression

By leveraging genome-wide information from our meta-analysis, we used LD-score regression^24–27^ to estimate heritability of grip strength, to evaluate partitioned heritability by functional categories and to identify genetic correlation with other traits. LD-score regression was conducted using the summary statistics from the meta-analysis of discovery and replication. We used pre-calculated European LD scores and restricted SNPs to those found in HapMap Phase III to ensure good quality imputation. We conducted partitioned heritability analysis using 24 main annotations described by Finucane *et al*.^27^. Enrichment was defined as the proportion of SNP heritability explained divided by the proportion of SNPs in each functional category. We considered FDR ≤ 0.05 to indicate statistically significant enrichments. Genetic correlation was tested against all traits in LD Hub^26^ after SNP filtering based on allele frequency, imputation quality, outliers in effect sizes, and removing SNPs in MHC region. To identify significant genetic correlations, we set the P-value threshold by correcting for the 231 phenotypes that were available in LD Hub at the time of analysis (0.05/231).

### Mendelian Randomization

We performed two-sample MR, which estimates the causal effect by contrasting the SNP effects on the exposure with the SNP effects on the outcome in independent datasets. SNPs from the meta-analysis were used as instrumental variables and publicly available GWAS data for CHD^47^ and AF^48^ as an outcome. If the SNPs were not available in the outcome GWAS, we used proxies in high LD with the lead variants (r^2^ ≥ 0.8) defined using 1000 Genomes European sample data. The effect sizes of SNPs were standardized and the alleles from the exposure and outcome GWAS were harmonized to match the same effect allele. In addition to standard inverse-variance weighted (IVW) regression, we used several sensitivity analyses^49^: 1) we excluded SNPs with heterogeneous instrumental variable estimates (based on their contribution to Cochran’s *Q* statistic), 2) we excluded SNPs if their association with other traits was more significant than with grip strength, 3) we applied robust analysis methods including penalization methods, median-based methods, and Egger regression. Consistency of the causal estimates across all SNPs was evaluated with heterogeneity statistics and Egger regression were used to assess horizontal pleiotropy. Analyses were conducted with R-package *TwoSampleMR*^50^, *MendelianRandomization*^51^ and *gtx*. Power for MR analyses was estimated with an online tool created by Burgess^52^. Analyses were conducted with R (version 3.3.0).

### Data availability

The data reported in this paper are available via application directly to the UK Biobank (http://www.ukbiobank.ac.uk/). GWAS summary statistics are available in LD Hub (http://ldsc.broadinstitute.org/).

## Electronic supplementary material

Supplementary material
