# Supplementary material for: Biological Insights Into Muscular Strength: Genetic Findings in the UK Biobank
Source: Sci Rep. 2018 Apr 24;8:6451. doi: 10.1038/s41598-018-24735-y (PMC5915424; doi:10.1038/s41598-018-24735-y)
Supplement: Supplementary file 1 — Supplementary material [file 41598_2018_24735_MOESM1_ESM.pdf]

# Supplementary Material

## Biological Insights Into Muscular Strength: Genetic Findings in the UK Biobank

Emmi Tikkanen, Stefan Gustafsson, David Amar, Anna Shcherbina, Daryl Waggott, Euan A. Ashley, Erik Ingelsson

**Supplementary Table S1. Genetic loci associated with grip strength in discovery and replication datasets.**

| | | | | | | Discovery | | | | Replication | | |
|---|---|---|---|---|---|---|---|---|---|---|---|---|
| SNP | CHR | position (GRCh37) | Nearest gene | Effect allele | Other allele | EAF | Beta | Se | P-value | Beta | Se | P-value |
| rs1421085 | 16 | 53800954 | FTO | C | T | 0.4 | -0.004 | 0.0003 | 5.4e-38 | -0.004 | 0.0005 | 4.9e-22 |
| rs12928404 | 16 | 28847246 | ATXN2L | C | T | 0.41 | -0.003 | 0.0003 | 1.0e-25 | -0.002 | 0.0004 | 1.1e-07 |
| rs13107325 | 4 | 103188709 | SLC39A8 | T | C | 0.075 | -0.006 | 0.0006 | 4.4e-23 | -0.005 | 0.0008 | 2.0e-10 |
| rs10203386 | 2 | 25136866 | ADCY3 | A | T | 0.45 | -0.003 | 0.0003 | 1.6e-20 | -0.003 | 0.0004 | 2.7e-09 |
| rs543874 | 1 | 177889480 | ENSG00000254154 | G | A | 0.21 | -0.004 | 0.0004 | 3.8e-20 | -0.003 | 0.0005 | 3.0e-07 |
| rs77226405 | 18 | 58077260 | MC4R | A | C | 0.02 | 0.01 | 0.0011 | 5.4e-19 | 0.007 | 0.0016 | 2.1e-05 |
| rs6752706 | 2 | 629831 | TMEM18 | C | T | 0.82 | -0.004 | 0.0004 | 3.9e-18 | -0.003 | 0.0006 | 2.9e-09 |
| rs2744475 | 6 | 50784880 | TFAP2B | G | C | 0.29 | -0.003 | 0.0003 | 6.5e-17 | -0.003 | 0.0005 | 3.9e-09 |
| rs2814949 | 6 | 34656722 | C6orf106 | G | A | 0.36 | -0.003 | 0.0003 | 6.8e-17 | -0.002 | 0.0005 | 0.001 |
| rs10871777 | 18 | 57851763 | ENSG00000267620 | G | A | 0.24 | -0.003 | 0.0004 | 7.6e-17 | -0.003 | 0.0005 | 7.2e-08 |
| rs3821269 | 2 | 70698590 | TGFA | G | A | 0.5 | 0.002 | 0.0003 | 3.5e-15 | 0.002 | 0.0004 | 0.00038 |
| rs12225051 | 11 | 47571967 | CELF1 | T | C | 0.28 | 0.003 | 0.0004 | 7.2e-15 | 0.003 | 0.0005 | 2.2e-08 |
| rs62106258 | 2 | 417167 | LINC01874 | C | T | 0.048 | 0.006 | 0.0007 | 3.3e-14 | 0.007 | 0.001 | 1.6e-12 |
| rs3810291 | 19 | 47569003 | ZC3H4 | A | G | 0.68 | -0.002 | 0.0003 | 1.3e-13 | -0.002 | 0.0005 | 0.00038 |
| rs1243188 | 10 | 21908803 | MLLT10 | C | T | 0.3 | -0.003 | 0.0003 | 1.6e-13 | -0.002 | 0.0005 | 1.1e-06 |
| rs2431112 | 5 | 103931707 | RP11-6N13.1 | A | G | 0.44 | -0.002 | 0.0003 | 1.7e-13 | -0.002 | 0.0004 | 2.4e-06 |
| rs10788958 | 1 | 54040670 | GLIS1 | G | C | 0.65 | 0.002 | 0.0003 | 1.2e-12 | 0.002 | 0.0005 | 0.001 |
| rs78648104 | 6 | 50683009 | TFAP2D | C | T | 0.086 | 0.004 | 0.0006 | 1.3e-12 | 0.002 | 0.0008 | 0.0016 |
| rs660010 | 18 | 53310969 | TCF4 | C | G | 0.14 | 0.003 | 0.0004 | 1.8e-12 | 0.003 | 0.0006 | 9.6e-07 |
| rs13130484 | 4 | 45175691 | ENSG00000248744 | T | C | 0.43 | -0.002 | 0.0003 | 2.0e-12 | -0.002 | 0.0004 | 0.00021 |
| rs4549685 | 7 | 39326478 | POU6F2 | T | C | 0.33 | 0.002 | 0.0003 | 2.0e-12 | 0.002 | 0.0005 | 8.1e-06 |
| rs17391694 | 1 | 78623626 | RNFT1P2 | T | C | 0.14 | -0.003 | 0.0005 | 2.8e-12 | -0.003 | 0.0006 | 1.2e-06 |
| rs6499240 | 16 | 69686912 | NFAT5 | G | A | 0.59 | -0.002 | 0.0003 | 3.7e-12 | -0.002 | 0.0004 | 5.6e-08 |
| rs80135947 | 17 | 65836001 | BPTF | C | A | 0.2 | -0.003 | 0.0004 | 4.8e-12 | -0.003 | 0.0006 | 3.7e-09 |
| rs2430740 | 12 | 15041938 | C12orf60 | G | T | 0.39 | -0.002 | 0.0003 | 6.0e-12 | -0.002 | 0.0005 | 0.00039 |
| rs2260051 | 6 | 31591918 | PRRC2A | T | A | 0.56 | -0.002 | 0.0003 | 7.1e-12 | -0.002 | 0.0004 | 3.0e-08 |

| | | | | | | | | | | | | |
|---|---|---|---|---|---|---|---|---|---|---|---|---|
| rs1556659 | 10 | 130834698 | ENSG00000232985 | T | C | 0.38 | 0.002 | 0.0003 | 1.1e-11 | 0.002 | 0.0005 | 4.6e-06 |
| rs7165759 | 15 | 80988600 | ABHD17C | A | G | 0.3 | 0.002 | 0.0003 | 1.4e-11 | 0.002 | 0.0005 | 5.1e-06 |
| rs9257280 | 6 | 28833507 | HCG14 | T | C | 0.23 | 0.003 | 0.0004 | 2.6e-11 | 0.002 | 0.0005 | 0.0034 |
| rs6738113 | 2 | 135634581 | CCNT2-AS1 | A | G | 0.36 | 0.002 | 0.0003 | 4.6e-11 | 0.002 | 0.0005 | 7.7e-07 |
| rs10035289 | 5 | 153095918 | GRIA1 | G | A | 0.53 | 0.002 | 0.0003 | 9.0e-11 | 0.001 | 0.0004 | 0.0011 |
| rs147730268 | 12 | 123024476 | KNTC1 | T | G | 0.088 | 0.004 | 0.0006 | 9.6e-11 | 0.003 | 0.0008 | 0.00022 |
| rs72917544 | 2 | 175241566 | CIR1 | A | G | 0.19 | 0.003 | 0.0004 | 1.5e-10 | 0.003 | 0.0006 | 5.6e-06 |
| rs181617194 | 12 | 122011598 | KDM2B | C | T | 0.041 | 0.005 | 0.0009 | 5.4e-10 | 0.004 | 0.0012 | 0.0035 |
| rs6265 | 11 | 27679916 | BDNF | T | C | 0.19 | 0.002 | 0.0004 | 7.1e-10 | 0.002 | 0.0006 | 0.00061 |
| rs11236203 | 11 | 74378000 | POLD3 | G | T | 0.5 | -0.002 | 0.0003 | 8.4e-10 | -0.001 | 0.0004 | 0.0028 |
| rs2760975 | 6 | 32560771 | HLA-DRB1 | A | G | 0.17 | -0.003 | 0.0005 | 9.7e-10 | -0.003 | 0.0006 | 7.0e-06 |
| rs12367809 | 12 | 50256063 | ENSG00000257253 | T | C | 0.37 | -0.002 | 0.0003 | 1.2e-09 | -0.002 | 0.0005 | 1.7e-05 |
| rs4677611 | 3 | 71407890 | FOXP1 | T | C | 0.49 | 0.002 | 0.0003 | 1.3e-09 | 0.001 | 0.0004 | 0.0052 |
| rs11121529 | 1 | 10271688 | KIF1B | G | C | 0.11 | -0.003 | 0.0005 | 2.1e-09 | -0.003 | 0.0007 | 0.00022 |
| rs113230003 | 19 | 18460956 | PGPEP1 | A | G | 0.26 | 0.002 | 0.0004 | 2.2e-09 | 0.002 | 0.0005 | 1.6e-05 |
| rs2439823 | 10 | 99778226 | CRTAC1 | G | A | 0.55 | -0.002 | 0.0003 | 2.6e-09 | -0.002 | 0.0004 | 2.7e-06 |
| rs6759321 | 2 | 136322676 | R3HDM1 | T | G | 0.24 | 0.002 | 0.0004 | 2.7e-09 | 0.003 | 0.0005 | 9.6e-07 |
| rs11766468 | 7 | 2082604 | MAD1L1 | A | G | 0.15 | 0.003 | 0.0004 | 2.8e-09 | 0.003 | 0.0006 | 4.1e-06 |
| rs11689199 | 2 | 100820421 | LINC01104 | G | A | 0.4 | 0.002 | 0.0003 | 3.1e-09 | 0.001 | 0.0005 | 0.005 |
| rs2307111 | 5 | 75003678 | POC5 | C | T | 0.39 | 0.002 | 0.0003 | 3.1e-09 | 0.002 | 0.0005 | 0.00024 |
| rs2053682 | 5 | 170599327 | RANBP17 | A | C | 0.68 | -0.002 | 0.0003 | 3.6e-09 | -0.002 | 0.0005 | 2.6e-05 |
| rs2206929 | 20 | 50899693 | RP4-723E3.1 | C | T | 0.29 | 0.002 | 0.0003 | 5.4e-09 | 0.001 | 0.0005 | 0.0088 |
| rs7955910 | 12 | 122654382 | LRRC43 | T | G | 0.48 | -0.002 | 0.0003 | 5.5e-09 | -0.001 | 0.0004 | 0.00085 |
| rs11594905 | 10 | 77659733 | C10orf11 | A | G | 0.14 | -0.003 | 0.0005 | 6.3e-09 | -0.002 | 0.0006 | 0.0094 |
| rs2625387 | 11 | 43613426 | MIR129-2 | A | G | 0.61 | 0.002 | 0.0003 | 9.7e-09 | 0.001 | 0.0005 | 0.0029 |
| rs9383061 | 6 | 15477610 | JARID2 | A | C | 0.32 | 0.002 | 0.0003 | 1.0e-08 | 0.002 | 0.0005 | 0.00098 |
| rs286818 | 5 | 107433446 | FBXL17 | A | T | 0.17 | 0.002 | 0.0004 | 1.1e-08 | 0.002 | 0.0006 | 0.00062 |
| rs12311114 | 12 | 124460703 | ZNF664:FAM101A | A | C | 0.31 | -0.002 | 0.0003 | 1.2e-08 | -0.002 | 0.0005 | 8.0e-05 |
| rs1513475 | 3 | 35724256 | ARPP21 | C | T | 0.35 | 0.002 | 0.0003 | 1.4e-08 | 0.002 | 0.0005 | 0.00044 |

| | | | | | | | | | | | | |
|---|---|---|---|---|---|---|---|---|---|---|---|---|
| rs4692996 | 4 | 94992328 | RP11-363G15.2 | C | T | 0.4 | 0.002 | 0.0003 | 1.4e-08 | 0.001 | 0.0005 | 0.002 |
| rs10807138 | 6 | 34184938 | CYCSP55 | A | G | 0.8 | 0.002 | 0.0004 | 1.7e-08 | 0.002 | 0.0006 | 0.0014 |
| rs117642368 | 17 | 43399058 | MAP3K14 | C | G | 0.11 | -0.003 | 0.0005 | 2.3e-08 | -0.002 | 0.0007 | 0.0072 |
| rs12507029 | 4 | 80853827 | ANTXR2 | T | C | 0.64 | 0.002 | 0.0003 | 2.7e-08 | 0.001 | 0.0005 | 0.0052 |
| rs12599952 | 16 | 72034653 | PKD1L3 | A | G | 0.32 | 0.002 | 0.0003 | 3.1e-08 | 0.002 | 0.0005 | 5.5e-06 |
| rs61872990 | 10 | 118649443 | ENO4:KIAA1598 | C | T | 0.17 | -0.002 | 0.0004 | 3.2e-08 | -0.002 | 0.0006 | 0.00027 |
| rs12475388 | 2 | 27039792 | CDKN2AIPNLP2 | A | G | 0.49 | 0.002 | 0.0003 | 3.4e-08 | 0.001 | 0.0004 | 0.00091 |
| rs28620278 | 15 | 56959028 | ZNF280D | T | C | 0.4 | -0.002 | 0.0003 | 3.5e-08 | -0.002 | 0.0005 | 2.9e-05 |
| rs4952499 | 2 | 40381921 | SLC8A1-AS1 | T | C | 0.74 | -0.002 | 0.0004 | 4.5e-08 | -0.001 | 0.0005 | 0.0045 |

SNP associations ordered according to significance in discovery analysis.
SNP, single-nucleotide polymorphism; CHR, chromosome, EAF, effect allele frequency

**Supplementary Table S2. Functional annotation of replicated single-nucleotide polymorphisms (SNPs).**

| SNP | CHR | Position | Nearest Gene | Distance to nearest gene | function | CADD | RDB | eQTL[aligned direction*] | OMIM |
|---|---|---|---|---|---|---|---|---|---|
| rs1421085 | 16 | 53800954 | FTO | 0 | intronic | 14.77 | 5 | | Growth retardation, developmental delay, facial dysmorphism (FTO) |
| rs12928404 | 16 | 28847246 | ATXN2L | 0 | intronic | 0.002 | 1b | TUFM (Whole_Blood, Skin_Sun_Exposed_Lower_leg), SULT1A2 (Whole_Blood) | Combined oxidative phosphorylation deficiency 4 (TUFM) |
| rs13107325 | 4 | 103188709 | SLC39A8 | 0 | exonic | 34 | 5 | | Congenital disorder of glycosylation, type Iin (SLC39A8) |
| rs10203386 | 2 | 25136866 | ADCY3 | 0 | intronic | 4.996 | 5 | ADCY3 (Whole_Blood, Nerve_Tibial), DNAJC27 (Whole_Blood) | |
| rs543874 | 1 | 177889480 | ENSG00000254154 | 8443 | intergenic | 9.231 | 7 | | |
| rs77226405 | 18 | 58077260 | MC4R | | | NA | NA | | |
| rs6752706 | 2 | 629831 | TMEM18 | 37504 | intergenic | 5.877 | 6 | | |
| rs2744475 | 6 | 50784880 | TFAP2B | 1556 | intergenic | 3.794 | 5 | | Char syndrome, Patent ductus arteriosus 2 (TFAP2B) |
| rs2814949 | 6 | 34656722 | C6orf106 | 0 | intronic | 1.61 | 6 | UHRF1BP1 (Artery_Tibial, Cells_Transformed_fibroblasts, Adipose_Subcutaneous) | |
| rs10871777 | 18 | 57851763 | ENSG00000267620 | 12025 | intergenic | 0.967 | 6 | | |
| rs3821269 | 2 | 70698590 | TGFA | 0 | intronic | 2.824 | NA | TGFA (Testis) | Alstrom syndrome, Orofacial cleft-2 (TGFA) |
| rs12225051 | 11 | 47571967 | CELF1 | 0 | intronic | 3.125 | 5 | C1QTNF4 (Adipose_Subcutaneous, Skin_Sun_Exposed_Lower_leg), MADD (Artery_Tibial) | Myotonic dystrophy 1 (CELF1) |
| rs62106258 | 2 | 417167 | LINC01874 | 0 | downstream | 19.65 | 7 | | |

| | | | | | | | | | |
|---|---|---|---|---|---|---|---|---|---|
| rs3810291 | 19 | 47569003 | ZC3H4 | 0 | UTR3 | 5.093 | 5 | | |
| rs1243188 | 10 | 21908803 | MLLT10 | 0 | intronic | 1.58 | 7 | | Leukemia, acute myeloid (MLLT10) |
| rs2431112 | 5 | 103931707 | RP11-6N13.1 | 0 | ncRNA_intronic | 1.534 | NA | | |
| rs10788958 | 1 | 54040670 | GLIS1 | 0 | intronic | 9.244 | 5 | | |
| rs78648104 | 6 | 50683009 | TFAP2D | 0 | exonic | 12.97 | 4 | | |
| rs660010 | 18 | 53310969 | TCF4 | 0 | intronic | 0.072 | 7 | | Corneal dystrophy, Fuchs endothelial, 3, Pitt-Hopkins syndrome (TCF4) |
| rs13130484 | 4 | 45175691 | ENSG00000248744 | 122022 | intergenic | 6.336 | 7 | | |
| rs4549685 | 7 | 39326478 | POU6F2 | 0 | intronic | 2.552 | 7 | | Wilms tumor susceptibility-5 (POU6F2) |
| rs17391694 | 1 | 78623626 | RNFT1P2 | 12539 | intergenic | 2.61 | 1f | FUBP1 (Artery_Tibial, Esophagus_Mucosa, Skin_Sun_Exposed_Lower_leg) | |
| rs6499240 | 16 | 69686912 | NFAT5 | 0 | intronic | 0.681 | 6 | CLEC18A (Whole_Blood, Thyroid, Adipose_Subcutaneous) | |
| rs80135947 | 17 | 65836001 | BPTF | 0 | intronic | 0.031 | 6 | | Alzheimer disease (BPTF) |
| rs2430740 | 12 | 15041938 | C12orf60 | 0 | intronic | NA | NA | ERP27 (Skin_Sun_Exposed_Lower_leg, Thyroid), RP11-233G1.4 (Testis) | |
| rs2260051 | 6 | 31591918 | PRRC2A | 0 | intronic | NA | NA | | |
| rs1556659 | 10 | 130834698 | ENSG00000232985 | 76372 | intergenic | 4.304 | 5 | | |
| rs7165759 | 15 | 80988600 | ABHD17C | 0 | intronic | 7.572 | 4 | | |
| rs9257280 | 6 | 28833507 | HCG14 | 1100 | intergenic | 4.58 | 4 | TRIM27 (Whole_Blood, Esophagus_Mucosa, Muscle_Skeletal) | |
| rs6738113 | 2 | 135634581 | CCNT2-AS1 | 0 | ncRNA_intronic | 2.154 | 6 | TMEM163 (Testis, Esophagus_Muscularis, Artery_Tibial) | |

| | | | | | | | | | |
|---|---|---|---|---|---|---|---|---|---|
| rs10035289 | 5 | 153095918 | GRIA1 | 0 | intronic | 2.982 | 5 | | |
| rs147730268 | 12 | 123024476 | KNTC1 | 0 | intronic | 0.894 | 6 | | |
| rs72917544 | 2 | 175241566 | CIR1 | 0 | intronic | 12.16 | 6 | | |
| rs181617194 | 12 | 122011598 | KDM2B | 0 | intronic | 1.754 | 6 | | |
| rs6265 | 11 | 27679916 | BDNF | 0 | exonic | 24.1 | 6 | BDNF (Nerve_Tibial, Thyroid) | Central hypoventilation syndrome, congenital (BDNF) |
| rs11236203 | 11 | 74378000 | POLD3 | 0 | intronic | 1.664 | 5 | | |
| rs2760975 | 6 | 32560771 | HLA-DRB1 | 3146 | upstream | NA | NA | | |
| rs12367809 | 12 | 50256063 | ENSG00000257253 | 0 | ncRNA_intronic | 0.633 | 7 | FAIM2 (Testis) | |
| rs4677611 | 3 | 71407890 | FOXP1 | 0 | intronic | 4.89 | 3a | | Mental retardation with language impairment and with or without autistic features (FOXP1) |
| rs11121529 | 1 | 10271688 | KIF1B | 0 | UTR5 | 18.01 | 4 | KIF1B (Whole_Blood), PGD (Cells_Transformed_fibroblasts, Muscle_Skeletal) | Charcot-Marie-Tooth disease, type 2A1, Pheochromocytoma, Neuroblastoma, susceptibility to, 1 (KIF1B) |
| rs113230003 | 19 | 18460956 | PGPEP1 | 0 | intronic | 0.291 | 6 | | |
| rs2439823 | 10 | 99778226 | CRTAC1 | 0 | intronic | 0.3 | 5 | | |
| rs6759321 | 2 | 136322676 | R3HDM1 | 0 | intronic | 1.432 | 7 | DARS (Thyroid), MCM6 (Cells_Transformed_fibroblasts), UBXN4 (Pancreas) | Hypomyelination with brainstem and spinal cord involvement and leg spasticity (DARS), Lactase persistence/nonpersistence (MCM6) |
| rs11766468 | 7 | 2082604 | MAD1L1 | 0 | intronic | 3.622 | 4 | | Lymphoma, somatic, Prostate cancer, somatic (MAD1L1) |
| rs11689199 | 2 | 100820421 | LINC01104 | 4295 | intergenic | 4.346 | 5 | AFF3 (Artery_Aorta) | |

| | | | | | | | | | |
|---|---|---|---|---|---|---|---|---|---|
| rs2307111 | 5 | 75003678 | POC5 | 0 | exonic | 0.049 | 5 | ANKDD1B (Nerve_Tibial, Thyroid), GCNT4 (Testis) | |
| rs2053682 | 5 | 170599327 | RANBP17 | 0 | intronic | 14.6 | 7 | | |
| rs2206929 | 20 | 50899693 | RP4-723E3.1 | 0 | ncRNA_intronic | 0.935 | NA | ZFP64 (Cells_Transformed_fibroblasts, Artery_Tibial) | |
| rs7955910 | 12 | 122654382 | LRRC43 | 0 | intronic | 0.54 | 7 | LRRC43 (Nerve_Tibial) | |
| rs11594905 | 10 | 77659733 | C10orf11 | 0 | intronic | 16.59 | 4 | | Albinism, oculocutaneous, type VII (C10orf11) |
| rs2625387 | 11 | 43613426 | MIR129-2 | 10393 | intergenic | 2.083 | NA | HSD17B12 (Skin_Sun_Exposed_Lower_leg, Thyroid, Artery_Tibial) | |
| rs9383061 | 6 | 15477610 | JARID2 | 0 | intronic | 0.807 | 5 | | |
| rs286818 | 5 | 107433446 | FBXL17 | 0 | intronic | 4.126 | 7 | | |
| rs12311114 | 12 | 124460703 | ZNF664:FAM101A | 0 | intronic | 3.424 | 6 | CCDC92 (Heart_Left_Ventricle, Adipose_Subcutaneous), ZNF664 (Thyroid) | |
| rs1513475 | 3 | 35724256 | ARPP21 | 0 | intronic | 2.243 | 7 | ARPP21 (Artery_Tibial, Artery_Aorta) | |
| rs4692996 | 4 | 94992328 | RP11-363G15.2 | 46615 | intergenic | 0.646 | 7 | | |
| rs10807138 | 6 | 34184938 | CYCSP55 | 2278 | intergenic | 2.707 | 7 | | |
| rs117642368 | 17 | 43399058 | MAP3K14 | 4644 | intergenic | 1.227 | 4 | CRHR1 (Cells_Transformed_fibroblasts, Adipose_Subcutaneous, Lung) | |
| rs12507029 | 4 | 80853827 | ANTXR2 | 0 | intronic | 2.539 | 7 | | Hyaline fibromatosis syndrome (ANTXR2) |
| rs12599952 | 16 | 72034653 | PKD1L3 | 0 | upstream | 0.467 | 7 | DHODH (Artery_Tibial, Nerve_Tibial, Cells_Transformed_fibroblasts) | Miller syndrome (DHODH) |
| rs61872990 | 10 | 118649443 | ENO4:KIAA1598 | 0 | intronic | 2.942 | 6 | KIAA1598 (Whole_Blood, Cells_Transformed_fibroblasts) | |
| rs12475388 | 2 | 27039792 | CDKN2AIPNLP2 | 10245 | intergenic | 1.724 | 4 | KHK (Skin_Sun_Exposed_Lower_leg), MAPRE3 (Skin_Sun_Exposed_Lower_leg), | Fructosuria (KHK) |

| | | | | | | | | | | |
|---|---|---|---|---|---|---|---|---|---|---|
| | | | | | | | | | DPYSL5 (Brain_Cerebellum) | |
| rs28620278 | 15 | 56959028 | ZNF280D | 0 | exonic | 2.125 | 7 | | | |
| rs4952499 | 2 | 40381921 | SLC8A1-AS1 | 0 | ncRNA_intronic | 12.86 | 7 | | | |

* The direction of effect to gene expression after aligning risk increasing allele of GWAS and tested allele of eQTLs; "+" when the risk increasing allele increase the expression of the gene, "-" when the risk increasing allele decrease the expression of the gene.
CHR, chromosome; CADD, Combined Annotation Dependent Depletion; RBD, Regulome database score; NA, information not available for this SNP.

**Supplementary Table S3. Genetic loci associated with grip strength in meta-analysis.**

| SNP | CHR | position | Nearest gene | Effect allele | Other allele | EAF | Beta | Se | P-value |
|---|---|---|---|---|---|---|---|---|---|
| rs11642015 | 16 | 53802494 | FTO | T | C | 0.4023 | -0.004 | 0.0003 | 7.5e-49 |
| rs62037364 | 16 | 28868695 | SH2B1 | A | G | 0.4076 | -0.003 | 0.0003 | 2.2e-26 |
| rs13135092 | 4 | 103198082 | SLC39A8 | A | G | 0.9169 | 0.005 | 0.0005 | 3.2e-26 |
| rs10203386 | 2 | 25136866 | ADCY3 | A | T | 0.452 | -0.003 | 0.0003 | 1.6e-23 |
| rs35142762 | 2 | 636790 | TMEM18 | T | C | 0.8282 | -0.004 | 0.0004 | 5.4e-22 |
| rs539515 | 1 | 177889025 | SEC16B | A | C | 0.7924 | 0.003 | 0.0003 | 3.1e-21 |
| rs62107261 | 2 | 422144 | FAM150B | T | C | 0.9518 | -0.006 | 0.0007 | 7.8e-21 |
| rs2744475 | 6 | 50784880 | TFAP2B | C | G | 0.7084 | 0.003 | 0.0003 | 1.5e-20 |
| rs2726036 | 16 | 28347140 | NPIPB6 | A | C | 0.5991 | 0.003 | 0.0003 | 3.4e-20 |
| rs34633411 | 18 | 57854694 | MC4R | T | C | 0.2374 | -0.003 | 0.0003 | 2.1e-19 |
| rs11039266 | 11 | 47532395 | CELF1 | T | G | 0.7219 | -0.003 | 0.0003 | 2.7e-18 |
| rs8073510 | 17 | 65839210 | BPTF | A | G | 0.8045 | 0.003 | 0.0004 | 1.3e-16 |
| rs1243182 | 10 | 21916728 | MLLT10 | T | C | 0.3108 | -0.002 | 0.0003 | 3.5e-16 |
| rs11645565 | 16 | 69896248 | WWP2 | A | G | 0.4308 | -0.002 | 0.0003 | 7.1e-16 |
| rs2260051 | 6 | 31591918 | PRRC2A | A | T | 0.4425 | 0.002 | 0.0003 | 1.0e-15 |
| rs2431112 | 5 | 103931707 | NUDT12 | A | G | 0.4407 | -0.002 | 0.0003 | 2.2e-15 |
| rs9469899 | 6 | 34793124 | UHRF1BP1 | A | G | 0.3572 | -0.002 | 0.0003 | 2.5e-15 |
| rs1642294 | 18 | 53374913 | TCF4 | C | G | 0.145 | 0.003 | 0.0004 | 5.9e-15 |
| rs3771498 | 2 | 70720070 | TGFA | T | C | 0.5169 | 0.002 | 0.0003 | 1.4e-14 |
| rs4549685 | 7 | 39326478 | POU6F2 | T | C | 0.3305 | 0.002 | 0.0003 | 4.5e-14 |
| rs6711390 | 2 | 135629439 | ACMSD | T | C | 0.3604 | 0.002 | 0.0003 | 6.0e-14 |
| rs11873305 | 18 | 58049192 | MC4R | A | C | 0.9608 | -0.005 | 0.0007 | 8.9e-14 |
| rs1556659 | 10 | 130834698 | MGMT | T | C | 0.3832 | 0.002 | 0.0003 | 1.0e-13 |
| rs7165759 | 15 | 80988600 | ABHD17C | A | G | 0.2981 | 0.002 | 0.0003 | 1.3e-13 |
| rs62512210 | 8 | 116623548 | TRPS1 | T | C | 0.9142 | -0.004 | 0.0005 | 1.9e-13 |
| rs7571826 | 2 | 230623127 | DNER | T | C | 0.3275 | -0.002 | 0.0003 | 3.7e-13 |

| | | | | | | | | | |
|---|---|---|---|---|---|---|---|---|---|
| rs11671304 | 19 | 47564643 | TMEM160 | T | C | 0.6729 | -0.002 | 0.0003 | 4.1e-13 |
| rs72917544 | 2 | 175241566 | CIR1 | A | G | 0.1861 | 0.003 | 0.0004 | 9.3e-13 |
| rs13130484 | 4 | 45175691 | GNPDA2 | T | C | 0.4333 | -0.002 | 0.0003 | 1.0e-12 |
| rs2287226 | 12 | 14987096 | ART4 | A | G | 0.6019 | 0.002 | 0.0003 | 1.2e-12 |
| rs6759321 | 2 | 136322676 | R3HDM1 | T | G | 0.2402 | 0.002 | 0.0003 | 2.4e-12 |
| rs34517439 | 1 | 78450517 | GIPC2 | A | C | 0.1247 | -0.003 | 0.0004 | 2.5e-12 |
| rs563296 | 10 | 99772404 | CRTAC1 | A | G | 0.5598 | -0.002 | 0.0003 | 3.4e-12 |
| rs10788958 | 1 | 54040670 | GLIS1 | C | G | 0.3512 | -0.002 | 0.0003 | 3.7e-12 |
| rs10846071 | 12 | 15016236 | NA | T | C | 0.3949 | -0.002 | 0.0003 | 3.8e-12 |
| rs2760975 | 6 | 32560771 | HLA-DRB1 | A | G | 0.168 | -0.003 | 0.0004 | 5.1e-12 |
| rs11766468 | 7 | 2082604 | MAD1L1 | A | G | 0.1467 | 0.003 | 0.0004 | 7.8e-12 |
| rs113315602 | 6 | 32574575 | HLA-DRB1 | A | C | 0.9027 | 0.003 | 0.0005 | 9.1e-12 |
| rs245774 | 5 | 170530930 | RANBP17 | A | G | 0.2717 | 0.002 | 0.0003 | 1.2e-11 |
| rs12367809 | 12 | 50256063 | BCDIN3D | T | C | 0.3684 | -0.002 | 0.0003 | 1.2e-11 |
| rs147730268 | 12 | 123024476 | KNTC1 | T | G | 0.0883 | 0.003 | 0.0005 | 1.8e-11 |
| rs113230003 | 19 | 18460956 | PGPEP1 | A | G | 0.2596 | 0.002 | 0.0003 | 1.9e-11 |
| rs2213581 | 6 | 32388546 | BTNL2 | T | C | 0.2346 | 0.002 | 0.0003 | 2.4e-11 |
| rs7755875 | 6 | 28854059 | TRIM27 | A | G | 0.3193 | 0.002 | 0.0003 | 2.6e-11 |
| rs12910459 | 15 | 56998196 | ZNF280D | T | C | 0.4326 | -0.002 | 0.0003 | 3.1e-11 |
| rs2126165 | 5 | 153167595 | GRIA1 | A | G | 0.4889 | -0.002 | 0.0003 | 3.8e-11 |
| rs41268905 | 6 | 32097611 | FKBPL | A | G | 0.1048 | -0.003 | 0.0005 | 4.1e-11 |
| rs56068671 | 6 | 50597378 | TFAP2D | T | G | 0.0832 | 0.003 | 0.0005 | 5.5e-11 |
| rs12599952 | 16 | 72034653 | DHODH | A | G | 0.3217 | 0.002 | 0.0003 | 7.3e-11 |
| rs62253653 | 3 | 53013267 | SFMBT1 | A | G | 0.7036 | -0.002 | 0.0003 | 9.5e-11 |
| rs4575361 | 12 | 124410529 | CCDC92 | A | T | 0.689 | 0.002 | 0.0003 | 1.2e-10 |
| rs879620 | 16 | 4015729 | ADCY9 | T | C | 0.6152 | -0.002 | 0.0003 | 1.2e-10 |

| | | | | | | | | | |
|---|---|---|---|---|---|---|---|---|---|
| rs1454687 | 3 | 94038085 | DHFRL1 | C | G | 0.4846 | -0.002 | 0.0003 | 1.9e-10 |
| rs11121542 | 1 | 10393920 | KIF1B | A | G | 0.122 | -0.003 | 0.0004 | 2.8e-10 |
| rs34916901 | 6 | 26286149 | HIST1H4H | T | G | 0.5116 | 0.002 | 0.0003 | 3.2e-10 |
| rs3847479 | 10 | 118833263 | NA | A | G | 0.1744 | -0.002 | 0.0004 | 4.2e-10 |
| rs12691307 | 16 | 29939877 | KCTD13 | A | G | 0.4398 | 0.002 | 0.0003 | 5.6e-10 |
| rs62004866 | 15 | 74223118 | LOXL1 | C | G | 0.8865 | 0.003 | 0.0004 | 5.6e-10 |
| rs12195114 | 6 | 26708207 | NA | A | G | 0.7513 | -0.002 | 0.0003 | 6.5e-10 |
| rs35511257 | 6 | 32545392 | HLA-DRB1 | C | G | 0.0956 | -0.003 | 0.0005 | 7.2e-10 |
| rs2237149 | 6 | 15412065 | JARID2 | A | C | 0.4131 | 0.002 | 0.0003 | 7.5e-10 |
| rs34341 | 5 | 74934009 | ANKDD1B | A | T | 0.4242 | 0.002 | 0.0003 | 8.4e-10 |
| rs11030104 | 11 | 27684517 | BDNF | A | G | 0.7964 | -0.002 | 0.0003 | 8.7e-10 |
| rs115131074 | 7 | 69238493 | AUTS2 | A | T | 0.0481 | -0.004 | 0.0007 | 9.0e-10 |
| rs72977282 | 11 | 74300441 | POLD3 | A | T | 0.4132 | -0.002 | 0.0003 | 9.0e-10 |
| rs73728611 | 6 | 32589363 | HLA-DQA1 | A | G | 0.8765 | 0.003 | 0.0004 | 9.9e-10 |
| rs181617194 | 12 | 122011598 | KDM2B | T | C | 0.9588 | -0.005 | 0.0008 | 1.2e-09 |
| rs7955910 | 12 | 122654382 | LRRC43 | T | G | 0.4825 | -0.002 | 0.0003 | 1.4e-09 |
| rs1513475 | 3 | 35724256 | ARPP21 | T | C | 0.6516 | -0.002 | 0.0003 | 1.5e-09 |
| rs40067 | 5 | 107439012 | FBXL17 | A | G | 0.1702 | 0.002 | 0.0004 | 1.6e-09 |
| rs4776614 | 15 | 73082468 | ADPGK | C | G | 0.3268 | 0.002 | 0.0003 | 1.9e-09 |
| rs7249 | 19 | 18391171 | JUND | T | C | 0.3669 | 0.002 | 0.0003 | 2.0e-09 |
| rs1951716 | 14 | 94007075 | UNC79 | T | C | 0.634 | -0.002 | 0.0003 | 2.4e-09 |
| rs62442203 | 7 | 28829253 | CREB5 | C | G | 0.4039 | 0.002 | 0.0003 | 2.7e-09 |
| rs58383713 | 4 | 80796055 | ANTXR2 | A | C | 0.196 | -0.002 | 0.0004 | 3.1e-09 |
| rs11223444 | 11 | 133221928 | OPCML | A | G | 0.4476 | -0.002 | 0.0003 | 3.2e-09 |
| rs4677611 | 3 | 71407890 | FOXP1 | T | C | 0.4877 | 0.002 | 0.0003 | 3.2e-09 |
| rs10807136 | 6 | 34178558 | HMGA1 | T | C | 0.1197 | -0.003 | 0.0004 | 3.3e-09 |

| | | | | | | | | | |
|---|---|---|---|---|---|---|---|---|---|
| rs1627854 | 2 | 26953540 | KCNK3 | A | G | 0.528 | 0.002 | 0.0003 | 3.5e-09 |
| rs202941 | 14 | 27686205 | NOVA1 | T | C | 0.6156 | 0.002 | 0.0003 | 4.2e-09 |
| rs12447992 | 16 | 64518662 | CDH11 | A | C | 0.8651 | -0.002 | 0.0004 | 4.7e-09 |
| rs76808502 | 2 | 145631179 | ZEB2 | C | G | 0.0291 | 0.005 | 0.0009 | 5.2e-09 |
| rs117108573 | 6 | 32489679 | HLA-DRB5 | T | C | 0.0816 | -0.003 | 0.0005 | 5.6e-09 |
| rs11689199 | 2 | 100820421 | AFF3 | A | G | 0.5978 | -0.002 | 0.0003 | 5.7e-09 |
| rs4755720 | 11 | 43628749 | HSD17B12 | T | C | 0.6084 | 0.002 | 0.0003 | 6.2e-09 |
| rs1039716 | 1 | 215566593 | KCTD3 | T | C | 0.7829 | 0.002 | 0.0003 | 6.5e-09 |
| rs10279237 | 7 | 69868489 | AUTS2 | T | C | 0.8003 | 0.002 | 0.0004 | 6.5e-09 |
| rs7658243 | 4 | 94995978 | SMARCAD1 | A | C | 0.5768 | -0.002 | 0.0003 | 6.6e-09 |
| rs529200 | 3 | 173114305 | NLGN1 | A | G | 0.4741 | 0.002 | 0.0003 | 6.7e-09 |
| rs61873510 | 10 | 102626510 | FAM178A | T | G | 0.3287 | -0.002 | 0.0003 | 7.7e-09 |
| rs4858934 | 3 | 88224304 | CGGBP1 | T | C | 0.1147 | 0.002 | 0.0004 | 8.7e-09 |
| rs3134986 | 6 | 32644553 | NA | T | C | 0.2899 | -0.002 | 0.0003 | 9.1e-09 |
| rs66679256 | 4 | 18351898 | LCORL | T | C | 0.4461 | -0.002 | 0.0003 | 9.2e-09 |
| rs13132853 | 4 | 38680015 | KLF3 | A | G | 0.6352 | -0.002 | 0.0003 | 9.3e-09 |
| rs207299 | 4 | 19096263 | LCORL | T | C | 0.2738 | 0.002 | 0.0003 | 9.6e-09 |
| rs61992671 | 14 | 101531854 | NA | A | G | 0.5063 | -0.002 | 0.0003 | 9.7e-09 |
| rs1523766 | 3 | 77670448 | ROBO2 | A | G | 0.4979 | -0.002 | 0.0003 | 1.0e-08 |
| rs17249398 | 4 | 102507114 | BANK1 | A | G | 0.0272 | -0.005 | 0.0009 | 1.2e-08 |
| rs77012907 | 2 | 24794214 | NCOA1 | A | G | 0.9098 | 0.003 | 0.0005 | 1.2e-08 |
| rs7231880 | 18 | 57967186 | MC4R | T | G | 0.7065 | 0.002 | 0.0003 | 1.2e-08 |
| rs1529883 | 15 | 47916872 | SEMA6D | C | G | 0.3453 | 0.002 | 0.0003 | 1.3e-08 |
| rs3113767 | 4 | 181536946 | TENM3 | T | C | 0.845 | 0.002 | 0.0004 | 1.3e-08 |
| rs6904571 | 6 | 40349366 | LRFN2 | T | C | 0.2588 | 0.002 | 0.0003 | 1.3e-08 |
| rs887013 | 7 | 39427769 | POU6F2 | A | T | 0.6818 | 0.002 | 0.0003 | 1.4e-08 |

| | | | | | | | | | |
|---|---|---|---|---|---|---|---|---|---|
| rs9320823 | 6 | 98429337 | MMS22L | T | C | 0.3981 | 0.002 | 0.0003 | 1.4e-08 |
| rs9579775 | 13 | 20616557 | ZMYM2 | A | C | 0.8646 | 0.002 | 0.0004 | 1.4e-08 |
| rs9877408 | 3 | 81974558 | GBE1 | A | G | 0.5984 | 0.002 | 0.0003 | 1.4e-08 |
| rs17024393 | 1 | 110154688 | GNAT2 | T | C | 0.9742 | 0.005 | 0.0009 | 1.5e-08 |
| rs2206929 | 20 | 50899693 | ZFP64 | T | C | 0.7096 | -0.002 | 0.0003 | 1.5e-08 |
| rs11084110 | 19 | 52220077 | NA | T | C | 0.7068 | 0.002 | 0.0003 | 1.6e-08 |
| rs453325 | 6 | 29906313 | HLA-A | A | C | 0.7549 | -0.002 | 0.0003 | 1.6e-08 |
| rs5750673 | 22 | 39110124 | GTPBP1 | A | G | 0.2776 | -0.002 | 0.0003 | 1.7e-08 |
| rs805343 | 2 | 54193193 | PSME4 | T | C | 0.475 | -0.002 | 0.0003 | 1.7e-08 |
| rs12680855 | 8 | 95582655 | KIAA1429 | A | G | 0.6826 | -0.002 | 0.0003 | 1.8e-08 |
| rs10758232 | 9 | 33775652 | PRSS3 | C | G | 0.6653 | 0.002 | 0.0003 | 2.0e-08 |
| rs303760 | 18 | 21083738 | C18orf8 | T | C | 0.346 | -0.002 | 0.0003 | 2.0e-08 |
| rs329120 | 5 | 133861756 | JADE2 | T | C | 0.418 | 0.002 | 0.0003 | 2.2e-08 |
| rs3843540 | 7 | 99126640 | ZKSCAN5 | T | C | 0.8514 | -0.002 | 0.0004 | 2.3e-08 |
| rs17769552 | 17 | 43927290 | SPPL2C | A | G | 0.2235 | -0.002 | 0.0003 | 2.3e-08 |
| rs6905419 | 6 | 12945093 | PHACTR1 | T | C | 0.2814 | -0.002 | 0.0003 | 2.4e-08 |
| rs4886869 | 15 | 77799657 | HMG20A | A | G | 0.3902 | 0.002 | 0.0003 | 2.5e-08 |
| rs6424971 | 1 | 185110367 | TRMT1L | T | C | 0.4829 | 0.002 | 0.0003 | 2.5e-08 |
| rs73077175 | 3 | 49774658 | IP6K1 | A | G | 0.3329 | 0.002 | 0.0003 | 2.6e-08 |
| rs901850 | 3 | 4758586 | ITPR1 | T | G | 0.256 | 0.002 | 0.0003 | 2.9e-08 |
| rs9641509 | 7 | 113622594 | PPP1R3A | T | G | 0.6135 | 0.002 | 0.0003 | 3.1e-08 |
| rs4452060 | 19 | 45347911 | PVRL2 | A | C | 0.42 | 0.002 | 0.0003 | 3.3e-08 |
| rs2044469 | 2 | 174961488 | OLA1 | A | G | 0.6525 | 0.002 | 0.0003 | 3.3e-08 |
| rs117642368 | 17 | 43399058 | SPATA32 | C | G | 0.1073 | -0.002 | 0.0005 | 3.4e-08 |
| rs4952499 | 2 | 40381921 | SLC8A1 | T | C | 0.7403 | -0.002 | 0.0003 | 3.4e-08 |
| rs12055409 | 6 | 33801510 | MLN | A | G | 0.3428 | -0.002 | 0.0003 | 3.5e-08 |

| | | | | | | | | | |
|---|---|---|---|---|---|---|---|---|---|
| rs143373756 | 5 | 153467446 | MFAP3 | C | G | 0.9812 | -0.006 | 0.0011 | 3.7e-08 |
| rs13017251 | 2 | 44221160 | LRPPRC | A | G | 0.494 | 0.002 | 0.0003 | 3.8e-08 |
| rs74550407 | 18 | 58058029 | MC4R | A | T | 0.0375 | 0.004 | 0.0007 | 3.8e-08 |
| rs830643 | 3 | 71667063 | FOXP1 | A | G | 0.4138 | 0.002 | 0.0003 | 4.0e-08 |
| rs1521624 | 15 | 79412399 | RASGRF1 | A | C | 0.4716 | 0.002 | 0.0003 | 4.0e-08 |
| rs6428601 | 1 | 91231085 | BARHL2 | A | C | 0.4549 | 0.002 | 0.0003 | 4.3e-08 |
| rs1329733 | 9 | 92208227 | GADD45G | A | G | 0.5243 | 0.002 | 0.0003 | 4.3e-08 |
| rs12162265 | 19 | 13955161 | NANOS3 | A | G | 0.2384 | -0.002 | 0.0003 | 4.6e-08 |
| rs9968060 | 3 | 62471282 | CADPS | T | C | 0.6456 | -0.002 | 0.0003 | 4.7e-08 |

SNP, single-nucleotide polymorphism; CHR, chromosome, EAF, effect allele frequency

**Supplementary Table S4. Results from two-sample Mendelian Randomization analysis for grip strength and coronary heart disease.**

| | All SNPs | | Heterogenic SNPs removed | |
|---|---|---|---|---|
| **Method** | **OR (95% CI)** | **P-value** | **OR (95% CI)** | **P-value** |
| Simple median | 0.61 ( 0.51 , 0.74 ) | <0.0001 | 0.65 ( 0.53 , 0.78 ) | <0.0001 |
| Weighted median | 0.52 ( 0.43 , 0.63 ) | <0.0001 | 0.65 ( 0.54 , 0.79 ) | <0.0001 |
| Penalized weighted median | 0.52 ( 0.43 , 0.63 ) | <0.0001 | 0.64 ( 0.53 , 0.77 ) | <0.0001 |
| IVW | 0.59 ( 0.49 , 0.71 ) | <0.0001 | 0.69 ( 0.60 , 0.79 ) | <0.0001 |
| Penalized IVW | 0.59 ( 0.52 , 0.67 ) | <0.0001 | 0.66 ( 0.58 , 0.76 ) | <0.0001 |
| Robust IVW | 0.58 ( 0.49 , 0.68 ) | <0.0001 | 0.68 ( 0.59 , 0.78 ) | <0.0001 |
| Penalized robust IVW | 0.59 ( 0.51 , 0.68 ) | <0.0001 | 0.66 ( 0.58 , 0.76 ) | <0.0001 |
| MR-Egger | 0.36 ( 0.19 , 0.69 ) | 0.002 | 0.52 ( 0.31 , 0.87 ) | 0.013 |
| Penalized MR-Egger | 0.41 ( 0.26 , 0.65 ) | <0.0001 | 0.50 ( 0.31 , 0.82 ) | 0.006 |
| Robust MR-Egger | 0.41 ( 0.25 , 0.67 ) | <0.0001 | 0.50 ( 0.30 , 0.81 ) | 0.006 |
| Penalized robust MR-Egger | 0.41 ( 0.26 , 0.63 ) | <0.0001 | 0.49 ( 0.31 , 0.76 ) | 0.002 |

Abbreviations: SNP, single nucleotide polymorphism; IVW, inverse-variance weighted; MR, Mendelian Randomization; OR, odds ratio; CI, confidence interval.

**Supplementary Table S5. Results from two-sample Mendelian Randomization analysis for grip strength and atrial fibrillation.**

| | All SNPs | | Heterogenic SNPs removed | |
|---|---|---|---|---|
| **Method** | **OR (95% CI)** | **P-value** | **OR (95% CI)** | **P-value** |
| Simple median | 0.69 ( 0.53 , 0.89 ) | 0.005 | 0.75 ( 0.58 , 0.97 ) | 0.028 |
| Weighted median | 0.64 ( 0.49 , 0.82 ) | <0.0001 | 0.67 ( 0.52 , 0.86 ) | 0.002 |
| Penalized weighted median | 0.64 ( 0.50 , 0.83 ) | 0.001 | 0.67 ( 0.52 , 0.86 ) | 0.002 |
| IVW | 0.69 ( 0.57 , 0.84 ) | <0.0001 | 0.75 ( 0.62 , 0.90 ) | 0.003 |
| Penalized IVW | 0.74 ( 0.62 , 0.88 ) | <0.0001 | 0.77 ( 0.65 , 0.92 ) | 0.002 |
| Robust IVW | 0.71 ( 0.58 , 0.88 ) | 0.002 | 0.75 ( 0.62 , 0.91 ) | 0.005 |
| Penalized robust IVW | 0.73 ( 0.61 , 0.88 ) | 0.001 | 0.76 ( 0.63 , 0.91 ) | 0.004 |
| MR-Egger | 0.35 ( 0.17 , 0.70 ) | 0.003 | 0.40 ( 0.19 , 0.84 ) | 0.015 |
| Penalized MR-Egger | 0.32 ( 0.17 , 0.58 ) | <0.0001 | 0.42 ( 0.21 , 0.82 ) | 0.011 |
| Robust MR-Egger | 0.30 ( 0.12 , 0.76 ) | 0.011 | 0.38 ( 0.14 , 1.00 ) | 0.05 |
| Penalized robust MR-Egger | 0.28 ( 0.11 , 0.71 ) | 0.007 | 0.39 ( 0.15 , 1.03 ) | 0.058 |

Abbreviations: SNP, single nucleotide polymorphism; IVW, inverse-variance weighted; MR, Mendelian Randomization; OR, odds ratio; CI, confidence interval.
